# Differential effects of hypoxia on etoposide-induced apoptosis according to the cancer cell lines

**DOI:** 10.1186/1476-4598-6-61

**Published:** 2007-09-26

**Authors:** Jean-Philippe Cosse, Audrey Sermeus, Kayleen Vannuvel, Noelle Ninane, Martine Raes, Carine Michiels

**Affiliations:** 1 Laboratory of Biochemistry and Cellular Biology (URBC), FUNDP-University of Namur, 61 rue de Bruxelles, 5000 Namur, Belgium

## Abstract

**Background:**

It is more and more recognized that hypoxia plays a role in the resistance of cancer cells to chemotherapy. However, the mechanisms underlying this resistance still need deeper understanding. The aim of this study was to investigate the effect of hypoxia on this process since hypoxia is one of the hallmarks of tumor environment.

**Results:**

The effect of hypoxia on the apoptosis induced by etoposide, one drug commonly used in chemotherapy, was investigated using three different cancer cell lines. Gene expression changes were also studied in order to delineate the mechanisms responsible for the hypoxia-induced chemoresistance. We observed that hypoxia differentially influenced etoposide-induced cell death according to the cancer cell type. While hypoxia inhibited apoptosis in hepatoma HepG2 cells, it had no influence in lung carcinoma A549 cells and further enhanced it in breast cancer MCF-7 cells. Etoposide increased p53 activity in all cell lines while hypoxia alone decreased it only in HepG2 cells. Hypoxia had no influence on the etoposide-induced p53 activity in A549, increased p53 abundance in MCF-7 cells but markedly decreased p53 activity in HepG2 cells. Using low density DNA arrays to detect the expression of genes involved in the regulation of apoptosis, etoposide and hypoxia were shown to each influence the expression of numerous genes, many of the ones influenced by etoposide being p53 target genes. Again, the influence of hypoxia on the etoposide-induced changes was different according to the cell type.

**Conclusion:**

These results evidenced that there was a striking parallelism between the effect of hypoxia on the etoposide-induced p53 stabilization as well as p53 target gene expression and its effect on the etoposide-induced apoptosis according to the cell type. They are very interesting not only because they provide one possible mechanism for the induction of chemoresistance under hypoxic conditions in cells like HepG2 but also because they indicate that not all cell types respond the same way. This knowledge is of importance in designing adequate treatment according to the type of tumors.

## Introduction

Chemotherapy is an integral component of standard care for solid tumors. However, recurrence may occur, with a poor clinical outcome. Some of the primary factors leading to chemoresistance begin to be understood. Overexpression of members of the ABC transporter family (MDR being the most well known), mutations as well as the development of therapeutic sanctuaries are well characterized to be responsible for drug resistance. Recently, the influence of hypoxia has also been recognized.

During tumor growth, the central area becomes hypoxic due to poor access to blood vessels capable of delivering oxygen [1,2]. Hypoxic regions have been evidenced in a wide range of cancers [3,4]. Low tumor oxygenation has been identified as an independent negative prognostic factor [5,6] and is associated with a higher risk of metastatic spread. In addition, hypoxia contributes to resistance to radiation therapy and to chemotherapy [7]. Hypoxia may directly induce tumor resistance via deprivation of molecular oxygen needed for some drugs to induce DNA damage. Indirectly, hypoxia may lead to treatment resistance by modulating gene expression resulting in resistance to cell death.

Many of the changes in gene expression observed under hypoxia are controlled by hypoxia-inducible factor-1 (HIF-1), a transcription factor specifically activated by oxygen deprivation [8]. HIF-1 is composed of two subunits belonging to the bHLH-PAS family: ARNT which is constitutively expressed in the nucleus and HIF-1α which is regulated by hypoxia. In normoxia (20% oxygen), HIF-1α is hydroxylated on two prolines (residues 564 and 402) by an oxygen-dependent prolyl hydroxylase and on the asparagine 803 by one oxygen-dependent asparaginyl hydroxylase, FIH-1. The two hydroxylated prolines are recognized by the protein pVHL, which is part of an ubiquitin ligase complex, thus targeting the HIF-1α subunit for degradation by the proteasome [9,10]. The hydroxylation of the asparagine prevents HIF-1α-CBP/p300 interaction [11]. In low oxygen conditions, HIF-1α is no longer modified and is thus stabilized. HIF-1α then translocates into the nucleus where it dimerizes with ARNT. The active HIF-1 binds to its specific site called HRE (hypoxia response element), present in the promoter of target genes. The products of these target genes (glucose transporter-1, VEGF and most of the glycolytic enzymes) allow the cell to adapt to the low oxygen conditions.

If mild hypoxia is rather pro-survival, it must be noted however that severe or prolonged hypoxia can lead to cell death, mainly through an apoptotic pathway [12,13]. HIF-1 seems to play a major role in this process by inducing p53 stabilization [14,15], overexpression of pro-apoptotic proteins such as BNIP3 [16] or HGTD-P [17] as well as Bax translocation [18].

It is thus apparent that hypoxia can either initiate apoptosis and cell death or prevent cell death by provoking adaptive response facilitating cell proliferation and tumor growth [4]. Considering that HIF-1 both induces the expression of pro-survival and cell death inducing genes, it is thus crucial to understand the fine tuning regulation that makes decision between life and death. Similarly, the influence of hypoxia on apoptosis resistance to radio- and chemotherapy still needs deeper understanding. The aim of this study was (i) to investigate the effect of hypoxia on the apoptosis induced by a drug used in chemotherapy using three different cancer cell lines and (ii) to define gene expression changes for each of them, in response to this drug, under normoxic and hypoxic conditions. Gene expression patterns were then correlated with the activity of HIF-1 and p53 in order to define molecular pathways involved in the cellular response as well as with the apoptotic profile observed under hypoxic conditions. We used etoposide as the apoptosis inducer. Etoposide is a topoisomerase II inhibitor that induces double strand breaks in DNA, thus leading to the activation of p53 and apoptosis [19]. We observed that hypoxia differentially influenced etoposide-induced cell death according to the cell type and that p53 may play a role in this process.

## Results

### Hypoxia differently modulates the etoposide-induced apoptosis according to the cell line

**Figure 1:**
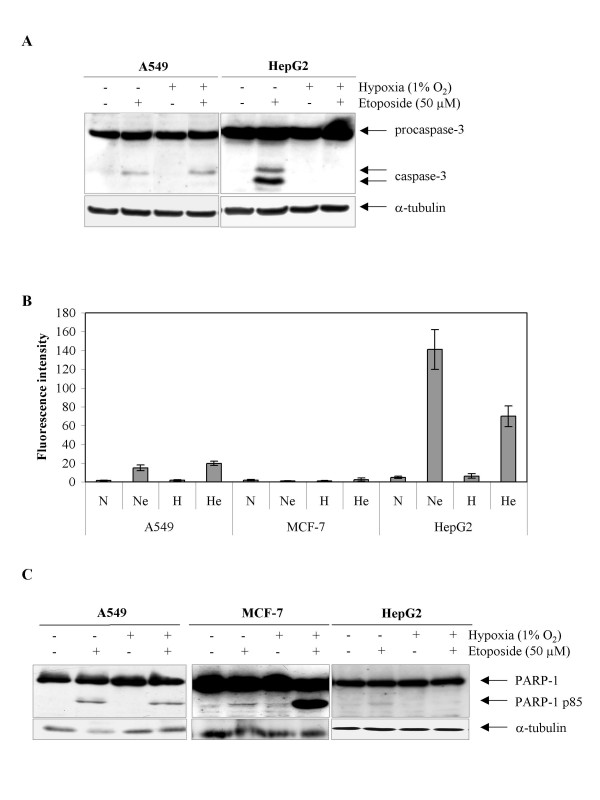
Effect of hypoxia on the etoposide-induced apoptosis. A549, MCF-7 or HepG2 cells were incubated under normoxic (N) or hypoxic (H) conditions with or without etoposide (e, 50 μM) for 16 hours.*A*, procaspase 3 and the 20 kDa long subunit were detected in total cell extracts by western blotting, using a specific anti-caspase 3 antibody. a-tubulin was used to assess the total amount of proteins loaded on the gel. *B*, the caspase 3 activity was assayed by measuring free AFC released from the cleavage of the caspase 3 substrate Ac-DEVD-AFC. Results are expressed in fluorescence intensity, as means ± 1 SD (n = 3). *C*, PARP-1 and cleaved 85 kDa fragment were detected in total cell extracts by western blotting, using a specific anti-PARP-1 antibody. a-tubulin was used to assess the total amount of proteins loaded on the gel.

Etoposide is known to induce apoptosis through DNA damage induced p53 activation [19]. We recently evidenced that hypoxia could protect HepG2 cells from the etoposide-induced apoptosis [20]. We then wanted to investigate whether such an effect was also observed using other cancer cell lines. We thus compared the effect of hypoxia on three cancer cell lines originating from different organs, all of them containing wild-type p53 : HepG2 from liver, A549 from lung and MCF-7 from breast. It has to be mentioned that MCF-7 cells are deficient for caspase 3, the main executive caspase. All three cell lines, incubated in the presence of 50 μM etoposide during 16 hours, did undergo apoptosis as shown by the appearance of the cleaved active form of caspase 3 (as observed by western blot), an increase in caspase 3 activity and in PARP cleavage (Fig. 1). Hypoxia alone did not induce apoptosis since no increase in any of these parameters was observed after 16 hours incubation. On the other hand, in HepG2, as already published [20], hypoxia inhibited the etoposide-induced apoptosis: a marked decrease in caspase 3 cleavage, in caspase 3 activity and in PARP cleavage was observed (Fig. 1). However, such a protective effect was not observed for the other cell lines: hypoxia did not influence etoposide-induced apoptosis in A549 cells while it seemed to enhance it in MCF-7 cells. Indeed, the abundance of PARP cleaved fragment was much higher when MCF-7 cells were incubated in the presence of etoposide under hypoxia than under normoxia (Fig. 1C).

**Figure 2:**
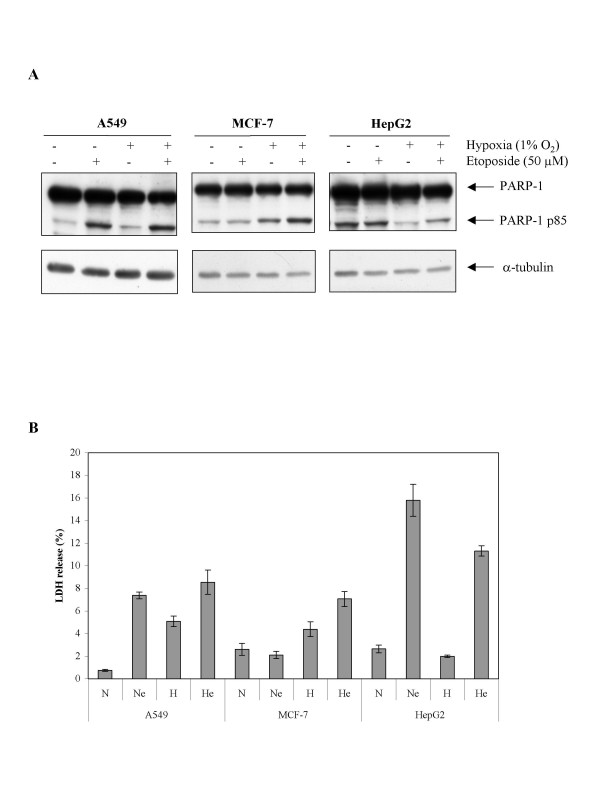
Effect of hypoxia on the etoposide-induced apoptosis. A549, MCF-7 or HepG2 cells were incubated under normoxic (N) or hypoxic (H) conditions with or without etoposide (e, 50 μM) for 40 hours. *A*, PARP-1 and cleaved 85 kDa fragment were detected in total cell extracts by western blotting, using a specific anti-PARP-1 antibody. a-tubulin was used to assess the total amount of proteins loaded on the gel. *B*, LDH release was assessed. Results are presented in percentages, as means ± 1 SD (n = 3).

Cell apoptosis and cell death were followed for a longer period of incubation in order to investigate whether the effect of hypoxia is sustained. Cells were incubated in the presence of etoposide under normoxic or hypoxic conditions for 40 h (Fig. 2). As an indication for apoptosis, PARP cleavage was assessed by western blot. Results (Fig. 2A) show that PARP cleavage induced by etoposide was more pronounced after 40 h incubation than after 16 h, in all three cell lines. Hypoxia prevented etoposide-induced PARP cleavage in HepG2 cells while it enhanced it in MCF-7 cells and had no effect in A549 cells. Cell death was also detected, as evidenced by an increase in LDH release, but again hypoxia differently influenced etoposide-induced cell death according to the cell type, with similar effects as observed for PARP cleavage. It has to be noted that hypoxia also induced a slight increase in cell death after 40 h incubation in A549 and MCF-7 cells. All together, these results indicate that the effect of hypoxia was sustained and translated in effect on cell viability.

### Hypoxia does not influence etoposide-induced DNA damage

Several pathways can influence apoptosis. Since etoposide induces DNA damage which then turns on p53, we first studied whether hypoxia could influence the induction of DNA damage by etoposide or could modulate the rate of DNA repair. As a surrogate for DNA damage, we followed phosphorylation on serine 139 of the histone H2AX [21], by immunofluorescence labeling using an antibody specific for the phosphorylated form of this histone. Cells were labeled 1 hour after the beginning of this incubation to evidence the induction of DNA damage by etoposide and after 16 hour incubation to assess DNA repair. Results shows that etoposide did induce histone H2AX phosphorylation in all three cell lines with HepG2 cells being the most sensitive [see Additional file 1]. DNA damage were still present after 16 hour incubation : the intensity of the labeling seemed weaker than after 1 hour incubation, for all three cell lines, suggesting DNA repair. Hypoxia did not influence the effect of etoposide nor the induction of DNA damage nor their repair.

### Etoposide does not affect HIF-1 activation under hypoxia

**Figure 3:**
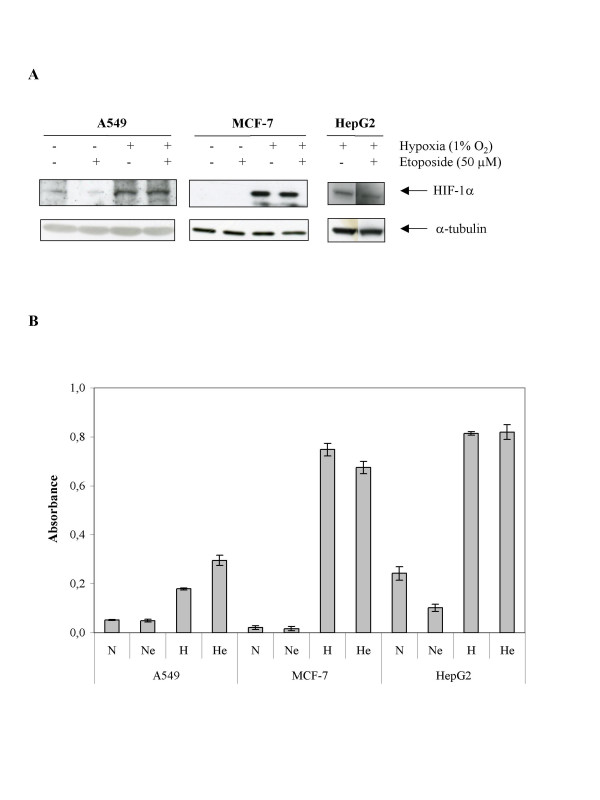
Effect of hypoxia and/or etoposide on the HIF-1α protein level and HIF-1 DNA binding activity. A549, MCF-7 or HepG2 cells were incubated under normoxic (N) or hypoxic (H) conditions with or without etoposide (e, 50 μM) for 16 hours. *A*, HIF-1α was detected in total cell extracts by western blotting. a-tubulin was used to assess the total amount of proteins loaded on the gel. *B*, after the incubation, nuclear extracts were performed from three independent experiments and hybridized in the ELISA well containing specific DNA probes (TransAM assay). Detection was performed using an anti-HIF-1α antibody. Results are expressed in absorbance (means ± 1 SD, n = 3).

HIF-1 has been shown to be responsible for an adaptive response of cells to hypoxia. If etoposide would influence its activity under hypoxia, this could lead to changes in cell survival. In order to investigate this possibility, we measured HIF-1α protein level by western blot and HIF-1 DNA binding activity. The results indicate that hypoxia did increase HIF-1α protein level and HIF-1 DNA binding activity in all three cell lines and these effects were not or very slightly influenced by etoposide (Fig. 3). It is thus not through modulation of HIF-1 activity that hypoxia affects etoposide-induced apoptosis according to the cell line.

### Hypoxia influences p53 activity differently according to the cell line

Etoposide induces cell death through the activation of p53, which then enhances the expression of pro-apoptotic genes. Hypoxia is known to modulate p53 protein level and activity [22]. However, this effect can be different according to the severity of hypoxia i.e. more severe hypoxia leads to p53 stabilization [14] while mild hypoxia does not. Decreased p53 protein level and activity has also been described for example in HepG2 cells exposed to 1 % O_2 _[23]. In order to investigate whether hypoxia would differently influence etoposide-induced p53 activation in the three cell lines used here, we studied the effect of hypoxia on p53 protein level as well as on its activity.

**Figure 4:**
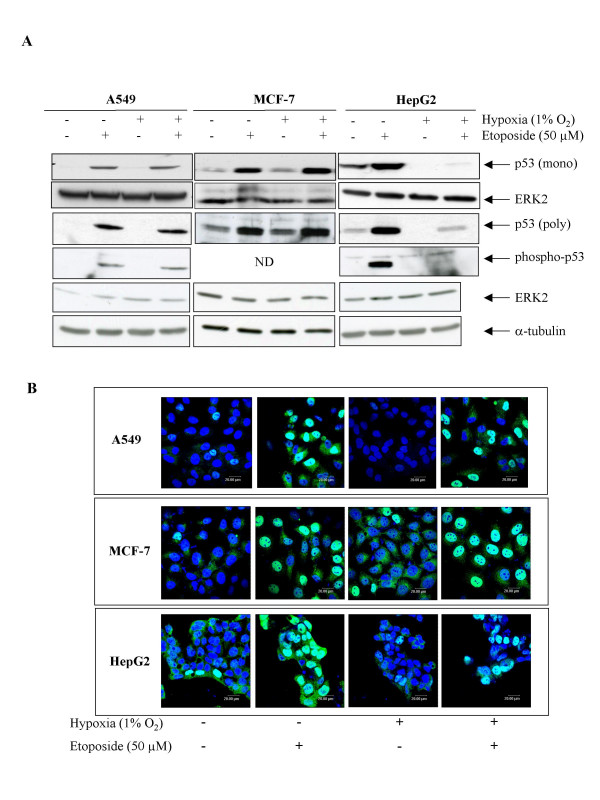
Effect of hypoxia on the etoposide-induced p53 stabilization. A549, MCF-7 or HepG2 cells were incubated under normoxic or hypoxic conditions with or without etoposide (50 μM) for 1 or 16 hours. *A*, p53 was detected in total cell extracts by western blotting, using two specific anti-p53 antibodies (a monoclonal and a polyclonal). Phosphorylation of p53 on serine15 was revealed using a specific antibody. ERK2 and a-tubulin were used to assess the total amount of proteins loaded on the gel. ND = non detected *B*, after the incubation, cells were fixed, permeabilized and stained for p53 using a specific antibody (green). Nuclei were detected with To-Pro-3 (blue). Observation was performed using a confocal microscope with the photomultiplier constant.

**Figure 5:**
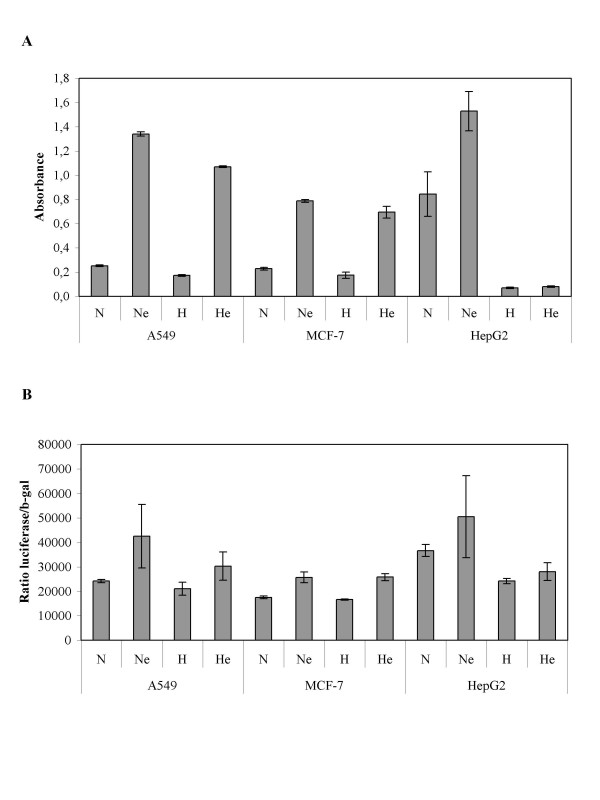
Effect of hypoxia on the etoposide-induced p53 activation. A549, MCF-7 or HepG2 cells were incubated under normoxic or hypoxic conditions with or without etoposide (50 μM) for 1 or 16 hours. *A*, after the incubation, nuclear extracts were performed from three independent experiments and hybridized in the ELISA well containing specific DNA probes (TransAM assay). Detection was performed using an anti-p53 antibody. Results are expressed in absorbance (means ± 1 SD, n = 3). *B*, Cells were cotransfected with the reporter plasmid pG13-Luc encoding the firefly luciferase and the pCMVβ normalization plasmid before being incubated 16 hours under normoxia (N) or hypoxia (H) in the presence or absence of etoposide (e, 50 μM). Results are expressed as means of the ratio between firefly luciferase activity and the β-galactosidase activity ± 1 SD (n = 3).

Figure 4 shows that in all three cell lines, etoposide induced an increase in p53 protein level as observed by western blot using two different antibodies (Fig. 4A) or immunofluorescence staining (Fig. 4B). It also increased p53 phosphorylation on serine 15 in A549 and HepG2 cells. No phosphorylation was detected in MCF-7 cells. p53 was mainly localized in the nucleus of stimulated cells. Hypoxia alone had little effect on p53 protein abundance in A549 and MCF-7 cells, which have a very low basal level of p53 under basal conditions. On the other hand, it markedly decreased p53 protein level in HepG2 cells. It has to be noted that, in this cell line, under basal condition, p53 is expressed at a high level and is mainly cytosolic. Moreover, hypoxia differentially affected the etoposide-induced increase in p53 protein level according to the cell type. It had no effect in A549 cells, induced a further increase in p53 protein abundance in MCF-7 but inhibited the effect of etoposide in HepG2 cells. There is a striking correlation between the effect of hypoxia on p53 protein level and its influence on etoposide-induced apoptosis. p53 DNA binding activity and actual transcriptional activity were also measured (Fig. 5A and 5B). Etoposide increased p53 activity in all cell lines while hypoxia alone decreased it only in HepG2 cells. Hypoxia had no influence on the etoposide-induced p53 activity in A549 and MCF-7 cells but markedly decreased it in HepG2 cells. These results are very similar to the ones obtained when assessing p53 protein level. The only difference is that hypoxia further enhanced the etoposide-induced increase in p53 protein abundance without repercussion on p53 DNA binding activity or transcriptional activity in MCF-7 cells. One possible explanation is that hypoxia influenced p53 modification needed for its stabilization but not all the post-translational modifications required for its activity (indeed we could not detected p53 phosphorylation on serine 15 in the presence of etoposide in MCF-7 cells) or that one co-factor became limiting.

**Figure 6:**
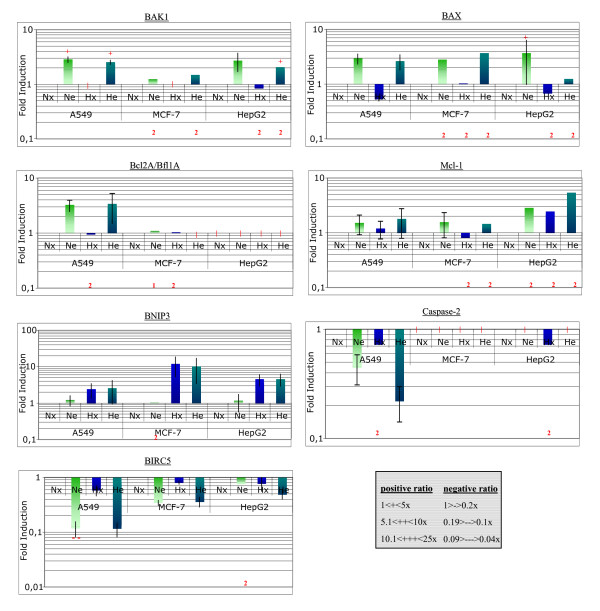
Gene expression profiling, for genes involved in regulating apoptosis, in A549, MCF-7 and HepG2 cells incubated with or without etoposide under normoxic or hypoxic conditions. Please refer to supplementary data [Additional file 2] for results obtained for the 62 genes for which there was a significant variation in expression for at least one of the conditions. Cells were incubated under normoxic (N) or hypoxic (H) conditions with or without etoposide (e, 50 μM) for 16 hours before RNA extraction, reverse-transcription and cDNA hybridization, as described in Materials and Methods. Each value is the average of three ratio values calculated from three independent experiments ± 1 S.D. Mean ratios indicate a fold-increase or decrease in gene expression. Qualitative values are given with + or - signs (according to the inserted table). The red vertical bars correspond to undetected cDNA. Duplicates or unique value are noted with a red 2 or 1 behind the corresponding column.

**Figure 7:**
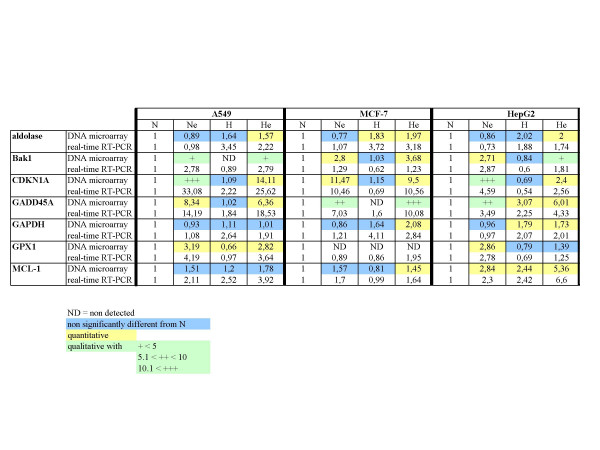
Comparison of the results obtained with real time RT-PCR and DNA microarrays analyses for *aldolase*, *BAK1, CDNK1A, GADD45A, GAPDH, GPX1*, and *MCL1 *genes. After the incubation, total RNA was extracted, submitted to reverse transcription and then to amplification in the presence of SYBR Green and specific primers. *RPL13A *was used as the house keeping gene for data normalization. For real-time RT-PCR results, data are given in fold-induction. For DNA microarray results, mean ratios indicate a fold-increase or decrease in gene expression. They are highlighted in blue if statistically non significant, in yellow for quantitative data and in green for qualitative data, given with + or - signs (according to the inserted table).

p53 DNA binding activity is mainly driven by p53 protein level while its transcriptional activity is regulated not only by p53 protein level but also by post-translational modifications like phosphorylation and acetylation. The western blot analysis shown in Figure 4A evidenced that hypoxia strongly decreased p53 protein level in HepG2 cells. There is a strong correlation between DNA binding activity and p53 protein level for HepG2 cells. The marked decrease in p53 protein level and DNA binding activity was not reflected in the luciferase activity assay, probably because there is already a quite high basal activity of the reporter system that is p53 independent (see normoxic control in A549 and MCF-7 while very low p53 protein level is detected by western blot) and that is not affected by hypoxia. The system can thus detect increased p53 activity as in the presence of etoposide but not diminished p53 activity. It has to be mentioned that hypoxia alone decreased the expression of several p53 target genes and decreased the etoposide overexpression of these genes in HepG2 cells (see results of figures 6 and 7). All together, these observations indicate that p53 activity is decreased under hypoxic conditions in HepG2 cells.

### Hypoxia and etoposide induce changes in gene expression

The apoptosis induced by etoposide is controlled by numerous pro- and anti-apoptotic genes. We hypothesized that hypoxia could induce gene expression alterations differentially according to the cell type, that would then influence the etoposide-induced apoptosis. We thus compared differences and similarities in gene expression modifications in both conditions i.e. etoposide under normoxic and hypoxic conditions in the three cell types. To investigate the expression of genes that are differentially regulated in these conditions, a low-density DNA microarray has been used. This microarray comprises 123 capture probes allowing gene expression analysis for a set of key genes related to apoptosis (DualChip^® ^human apoptosis, Eppendorf). Cells were incubated during 16 hours in the presence or in the absence of etoposide at 50 μM under normoxia or hypoxia. Cells were harvested and total RNA was extracted and reverse transcribed into cDNA using biotinylated nucleotides. The resulting cDNAs were hybridized on the microarray and revealed with a cyanin 3-conjugated anti-biotin antibody. Three independent experiments were performed for each condition and each sample was hybridized to three submicroarrays represented by the probes spotted in triplicates. The array data were subjected to a simple algorithm (see Materials and Methods) to set a lower threshold and to normalize the data using internal standard controls and house keeping genes. Then, average ratios compared to normoxia and their standard deviations (S.D.) were calculated. We ended up with a list of gene transcripts that were not detected due to their low abundance, detected with no modification, quantitatively up- or down-regulated and qualitatively up-or down-regulated. Genes were sorted in the latter category when the fluorescent signal was either completely saturated or lower than 2.5× the local background in one of the experimental conditions.

**Figure 8:**
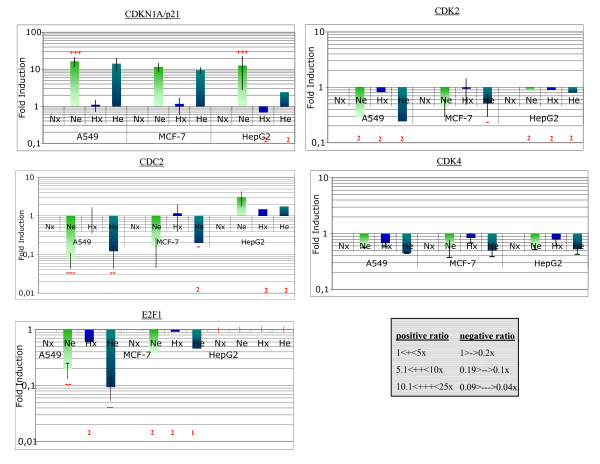
Gene expression profiling, for genes involved in regulating cell cycle, in A549, MCF-7 and HepG2 cells incubated with or without etoposide under normoxic or hypoxic conditions. Please refer to supplementary data [Additional file 2] for results obtained for the 62 genes for which there was a significant variation in expression for at least one of the conditions. Cells were incubated under normoxic (N) or hypoxic (H) conditions with or without etoposide (e, 50 μM) for 16 hours before RNA extraction, reverse-transcription and cDNA hybridization, as described in Materials and Methods. Each value is the average of three ratio values calculated from three independent experiments ± 1 S.D. Mean ratios indicate a fold-increase or decrease in gene expression. Qualitative values are given with + or - signs (according to the inserted table). The red vertical bars correspond to undetected cDNA. Duplicates or unique value are noted with a red 2 or 1 behind the corresponding column.

All the changes in gene expression are presented in the supplementary table [see Additional file 2]. Some of the genes upregulated by etoposide, are genes known to be p53 target genes (*GADD45, BAX, MDM2*) while some of the genes upregulated by hypoxia alone are genes known to be HIF-1 target genes (*BNIP3, ALDOA*). The increased expression of the genes induced by hypoxia did not seem to be affected by the presence of etoposide. These results are coherent with the fact that etoposide did not influence HIF-1 activity under hypoxia (see Fig. 3). Two families of genes for which changes in gene expression could be of interest were outlined. The first list identified 7 genes whose products are directly involved in switching on or off the apoptosis process (Fig. 6). Of note are *BAX *and *BAK *whose expression was significantly increased by etoposide in all cell lines. Moreover, hypoxia influenced this increase differentially according to the cell type: it had no effect on A549 cells, inhibited the etoposide-induced increased in *BAX *and *BAK *expression in HepG2 cells while further enhanced it in MCF-7 cells. These expression profiles are similar to the ones for p53 protein level and for apoptotic level. There are also genes upregulated both by etoposide and by hypoxia alone (e.g. *MCL1*) and the combined effect of both conditions was additive in HepG2 cells but not in the two other cell types. Finally, the expression of *BIRC5 *(encoding survivin) was decreased under hypoxic conditions, and to a much larger extent in the presence of etoposide. The effect of both was additive in HepG2 cells. The second list of genes includes genes whose product is involved in regulating cell cycle : in general, etoposide increased the expression of cell cycle inhibitors (e.g. *CDKN1A *which encodes p21) while decreasing the expression of genes coding for kinases or transcription factors needed for progression through the cell cycle (*CDK2, CDC2, CDK4, E2F1*) (Fig. 8). These observations are true for all three cell lines. In most cases, hypoxia alone did not influence the expression of these genes and did not influence the effect of etoposide.

The fact that our data are in good agreement with previous studies reporting modifications in HIF-1 target gene expression during hypoxia as well as in p53 target genes in the presence of etoposide already validates the DNA microarray used in this study. In order to further validate our data, we also performed SYBR Green quantitative real time PCR assays for some selected genes. Values obtained for a set of 7 genes that were up-regulated or down-regulated in response to at least one condition were confirmed (Fig. 7). For these genes, we found a very good correlation between relative transcript abundance data obtained by DNA microarray and by real time RT-PCR.

**Figure 9:**
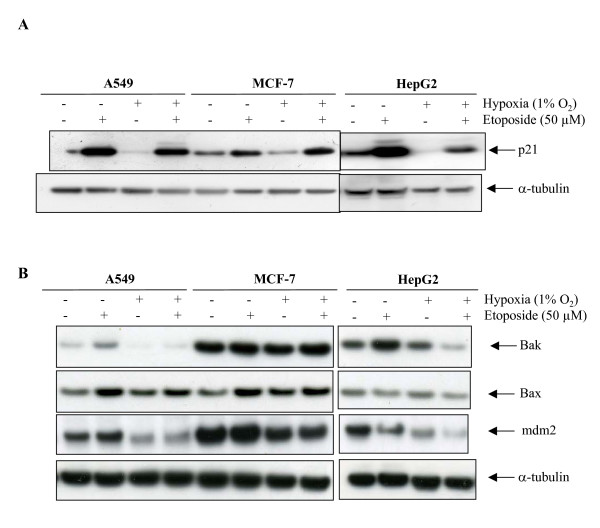
Effect of hypoxia and/or etoposide on p21 (A), Bak, Bax and mdm2 (B) protein levels. A549, MCF-7 or HepG2 cells were incubated under normoxic (N) or hypoxic (H) conditions with or without etoposide (e, 50 μM) for 16 hours. p21, Bak, Bax and mdm2 were detected in total cell extracts by western blotting, using specific antibodies. a-tubulin was used to assess the total amount of proteins loaded on the gel.

Protein levels of three p53 target genes (p21, Bax and mdm2) as well as of Bak were then assessed (Fig. 9). There is a very good correlation between *CDKN1A *mRNA and p21 protein level: etoposide markedly increased both in all three cell lines while hypoxia alone decreased both only in HepG2 cells. Hypoxia did not influence the etoposide-induced increase in p21 protein level in A549 cells, strongly decreased it in HepG2 cells and seemed to further enhance it in MCF-7 cells. Similar observations can be made for Bak, with a good correlation between mRNA and protein levels but not for Bax and mdm2. *BAX *mRNA expression profile is similar to the ones for *CDKN1A *and *BAK *mRNA. Bax protein levels followed mRNA abundance in A549 and MCF-7 cells but not in HepG2 cells. Indeed, in these cells, etoposide induced a decrease in Bax protein abundance while hypoxia had no effect. There is a correlation between all these results and the evolution of p53 protein level, but not with HIF-1α protein level. Finally, in all three cell lines, etoposide increased by several folds *mdm2 *mRNA level with no influence of hypoxia. On the other hand, an increase in mdm2 protein level in the presence of etoposide was observed in A549 and in MCF-7 cells while there was a decrease in HepG2 cells. Under hypoxic conditions, the protein was much less abundant in the three cell types even if an increase in mRNA level was observed in the presence of etoposide. All these results indicate that post-transcriptional events are involved in regulating mdm2 protein level similarly in the three cell lines while a regulation specific to the HepG2 cells is involved for the regulation of Bax, Bak and p21 expression.

## Discussion

Hypoxia is described to induce resistance of cancer cells to radio- and chemotherapy. In addition to decrease oxygen availability needed for radiation or for some of the chemical drugs to induce damage to the cells, hypoxia also modifies gene expression, thus rendering the cells less sensitive to these treatments. The exact mechanisms triggered by hypoxia that lead to resistance to apoptosis are not known, neither are the genes involved in this resistance that are turned on. The results presented in this work evidenced that not all cell types are protected by hypoxia. If the apoptosis induced by etoposide is inhibited in HepG2 cells, oxygen deficiency did not influence the etoposide-induced cell death in A549 cells and even further enhanced cell death induced in MCF-7 cells. Indeed, etoposide-induced increase in caspase 3 activity and PARP cleavage was differentially modulated by hypoxia according to the cell type. Etoposide triggers apoptosis through the induction of double strand DNA damage that then lead to p53 stabilization. Our results indicate that hypoxia does not influence the induction, nor the reparation of DNA damage. However, there was a striking parallelism between the effect of hypoxia on the etoposide-induced p53 stabilization and its effect on the etoposide-induced apoptosis according to the cell type.

Numerous reports have evidenced positive or negative effects of hypoxia on p53. The first one was the work of An et al [14] that showed that under severe hypoxic conditions, HIF-1α can directly interact with p53 leading to its stabilization. Further ones also showed that hypoxia also led to p53 stabilization probably through a displacement of p53 from mdm2 by HIF-1α. It must be noted that even if stabilized in these conditions, p53 is often not transcriptionally active [24]. On the other hand, other works showed no effect or even a decrease in p53 protein level under hypoxia [25] and a careful analysis of the experimental conditions used in the several studies revealed that p53 is stabilized under severe or prolonged hypoxia while mild hypoxia does not influence or even decreases p53 protein level [12,22]. Difference in HIF-1α phosphorylation status according to the severity of hypoxia could explain why it binds to p53 only in severe hypoxic conditions [26]. On the other hand, p53 phosphorylation by CK2 targeting this protein for degradation by the proteasome system in mild conditions has been proposed to explain its lower abundance in HepG2 cells under these conditions [23]. If it is known that according to the degree of hypoxia, opposite effects can be observed on p53 protein level, there has been no report until now evidencing opposite effects according to the cell type exposed to the same decrease in pO_2_, as we observed here. Chi et al [27] elegantly demonstrated that different cell types (endothelial or epithelial vs smooth muscle cells) respond differently to hypoxia through the induction of a different panel of hypoxic-responsive genes. However, no p53 responsive gene was described in their study. The mechanisms underlying the specific effects of hypoxia in cancer cells in relation to apoptosis remain to be investigated.

The differential effect of hypoxia on the etoposide-induced increase in p53 protein level in the three cell lines studied here is reflected on its activity. Indeed, in HepG2 cells, we observed a decrease in p53 DNA binding activity, in its transcriptional activity measured with the use of a reporter system and in the relative mRNA abundance of its target genes (e.g. *CDKN1A, BAX, BAK, GADD45, GPX1*), as measured using the DNA microarray. Similarly, further increase in the etoposide-induced in the expression of the mRNA for four of these target genes (*GPX1 *mRNA was not detected in MCF-7 cells) was evidenced in MCF-7 cells. The reason why such an increase was not observed using the reporter system is not known, but may involve a saturation of the luciferase expression or limiting amount of cofactor(s). Finally, hypoxia did not influence the etoposide-induced increase in p53 activity nor in the expression of its target genes in A549 cells.

All together, these results suggest that hypoxia inhibits or aggravates the etoposide-induced apoptosis through inhibition or further activation of the etoposide-induced increase in p53 transcriptional activity. p53 activity leads to the overexpression of pro-apoptotic genes like *BAX *and *BAK*, which can then be responsible for the induction of apoptosis. By modulating their expression, hypoxia directly modulates cell death. It has to be noted that protein expression does not always follow mRNA levels. If it was the case for Bak, Bax protein abundance in HepG2 involves post-transcriptional mechanisms. Secondly, pro-apoptotic gene transcription is not the only way by which p53 induces apoptosis. This protein can also triggers the intrinsic apoptotic pathway through direct protein-protein interaction notably with Bak and Bcl-2 [28]. Such a mechanism may also be switched on by hypoxia in MCF-7 cells. Indeed, immunofluorescence studies (Fig. 4) showed that, in these cells, p53 was not only localized in the nucleus but also in the cytoplasm. Further investigation is needed to confirm this hypothesis.

In addition to prevent p53-dependent transcription of Bax, hypoxia also interferes with Bax expression/function in a transcriptionally independent manner. Indeed, Kim et al [29] show that hypoxia blocked Bax translocation from the cytosol to the outer mitochondrial membrane, hence preventing TRAIL-induced apoptosis. Moreover, oxygen deprivation also results in a decreased Bax protein expression in different cell lines, in a proteasome-independent manner, probably through a reduction in global translation efficiency [30]. In this report, Bax decreased expression led to cell resistance to drug-induced apoptosis under hypoxia.

The regulation of p21 expression is complex. p21 mRNA has been described to be upregulated by DNA damaging agents and under hypoxic conditions probably through different mechanisms. Krieg et al [31] demonstrated that p53 binds to the p21 promoter in both conditions but is only active to enhance p21 gene expression in the presence of DNA damaging agents. p53 seems not to be responsible for the enhanced p21 expression under hypoxia. On the other hand, Koshiji et al [32] showed that p21 overexpression under hypoxia is due to HIF-1α : HIF-1α counteracts c-myc repression of p21 expression under these conditions. Both p53 and HIF-1α are thus probably involved in our experimental conditions, probably differently according to the conditions (etoposide versus hypoxia, or both together).

A clear correlation between the hypoxia-induced protection against etoposide-induced apoptosis and the effect of hypoxia on p53 was evidence in HepG2 cells in this work. It is thus probably by inhibiting the expression of p53-dependent pro-apoptotic genes that hypoxia is protective. These results are in good accordance with a recent study published by Wang et al [33] that shows that hypoxia could protect cisplatin-induced tubular cell apoptosis in a HIF-1-independent way but via suppression of p53 functions.

Etoposide modulates the expression of numerous genes at least in part through a p53-dependent mechanism. However, various changes in gene expression are also observed in p53-deficient cells as observed in HL-60 leukemic cells [34], indicating that other mechanisms are involved. In this study, etoposide induced a decrease in the expression of several cell cycle regulating genes. This decrease is similar in all the three cell lines and was not modulated by hypoxia, suggesting that it is probably independent of p53 activity.

The results of this study are very interesting not only because they provide one possible mechanism explaining chemoresistance under hypoxic conditions in cells like HepG2, but also because they shake up the idea that the presence of p53 is a good prognostic factor for the patient response to chemotherapy. Indeed, it does not always seem to be the case for example if hypoxic conditions are superimposed to the application of the chemotherapeutic drug since, in the case of HepG2 cells, it is its inhibition by hypoxia that leads to chemoresistance. A better understanding of the interplay between the apoptosis-inducing mechanisms and the pro-survival pathways initiated by hypoxia is thus needed for developing better therapeutic strategies to treat cancer patients.

## Methods

### Cell culture and hypoxia incubation

Human hepatoma cells HepG2, human breast adenocarcinoma MCF-7 and human pulmonary A549 cells were maintained in culture in 75-cm^2 ^polystyrene flasks (Costar) with respectively Dulbecco's modified Eagle's medium liquid (DMEM), Roswell Park Memorial Institute (RPMI) or modified Eagle's medium (MEM), containing 200 U/ml penicillin and 200 μg/ml streptomycin (Biowhittaker Europe) and 10% of foetal calf serum and incubated under an atmosphere containing 5% CO_2_.

For hypoxia experiments (1 % O_2_), cells were incubated in serum-free CO_2_-independent medium (Invitrogen) supplemented with 1 mM L-glutamine (Sigma) with or without etoposide (Sigma) at 50 μM. Normoxic control cells were incubated in the same conditions but in normal atmosphere (20 % O_2_).

### LDH release

LDH release was measured with the «cytotoxicity detection kit» from Roche Molecular Biochemical according to the manufacturer's protocol. The culture media from incubated cells were removed and centrifuged to pellet the cell fragments and apoptotic bodies. In order to lyse the cells, triton ×100 (Merck) at 10 % in DMEM was added on this pellet as well as on the cells remaining in the wells. The percentage LDH release was calculated as follows:

LDH activity in medium (1) + LDH activity of cell fragments (2)/(1) + (2) + LDH activity of cells remaining in the wells.

### Caspase 3 activity

The fluorogenic substrate Ac-DEVD-AFC was used to measure caspase 3 activity according to Lozano et al [35]. Cell extracts were prepared as described by Wellington et al [36]. Cells were seeded in 6-well plates (250,000 cells/well for HepG2 and A549 and 200,000 cells/well for MCF-7). After the incubation, the medium was recovered and centrifuged at 1,000 g for 5 min. Cells still attached to the well were scrapped in 200 μl cold PBS and recovered into a microtube. Pelleted detached cells were resuspended in 100 μl PBS at 4°C and also added to the microtube. The samples were centrifuged at 1,000 g for 5 min. at 4°C and the pellet resuspended in 50 μl of lysis buffer (10 mM Hepes/KOH, pH 7.0, 10 % sucrose, 2 mM EDTA, 0.1 % CHAPS, 5 mM dithiothreitol and 10 μg/ml aprotinin). After incubation at 4°C on a rotating wheel for 15 min, the lysates were centrifuged at 13,000 g for 5 min at 4°C and the supernatants were recovered for the assay. The protein concentration was measured and 5 μg completed to 50 μl with lysis buffer were mixed with 13 μM Ac-DEVD-AFC (BD Pharmingen) and 50 μl reaction buffer (40 mM PIPES, pH 7.2, 200 mM NaCl, 2 mM EDTA, 0.2 % CHAPS, 0.10 % sucrose and 10 mM dithiothreitol). The reaction was allowed to take place for 1 hour at 37°C and the fluorescence generated by the release of the fluorogenic group AFC on cleavage by caspase 3 was measured by excitation at 400 nm and emission at 505 nm.

### Western blotting

Cells, seeded in 25 cm^2 ^flasks, were scrapped in 200 μl of lysis buffer (Tris 40 mM pH 7.5, KCl 150 mM, EDTA 1 mM, triton X-100 1%) containing a protease inhibitor mixture («Complete» from Roche Molecular Biochemicals, 1 tablet in 2 ml H_2_O, added at a 1: 25 dilution) and phosphatase inhibitors (NaVO_3 _25 mM, PNPP 250 mM, α-glycerophosphate 250 mM and NaF 125 mM, at a 1: 25 dilution). Western blot analysis was performed as described in [20] using mouse anti-PARP-1 monoclonal antibody (#556493 from Pharmingen) as the primary antibody. Mouse anti-PARP-1 monoclonal antibody (#556493 from Pharmingen), mouse anti-p53 (#05–224 Upstate), rabbit anti-p53 (SC-6243 Santa Cruz), mouse anti-phosphorylated serine 15-p53 (#9286 Cell Signaling) and mouse anti-HIF-1α (# 610958 Becton Dickinson) were used at 1/5,000 dilution. Sheep anti-mouse or rabbit IgG horseradish peroxidase-linked antibody (Amersham Pharmacia Biotech) was used at 1/100,000 dilution as the secondary antibody.

For detection of caspase 3, Bax and p21, samples were applied to 4–12% NuPAGE Bis-Tris gels with MES buffer (Invitrogen), according to the manufacturer's instructions and then, transferred to Hybond-PVDF membrane (Amersham) or nitrocellulose membrane for detecting caspase 3 (BioRad). Membranes were blocked overnight at 4°C in TBS-Tween 2% blocking agent. Then, membranes were probed with rabbit anti-caspase 3 antibody (#9662 Cell Signaling), rabbit anti-p21 antibody (SC-398 Santa Cruz) or rabbit anti-Bax (#06–499 Upstate) at a final dilution 1/5,000 overnight at 4°C. After 3 × 15 min washes in TBS-Tween, the incubation with the secondary antibody was performed for 45 min in TBS-Tween 2% blocking agent followed by 3 washes of 15 min in TBS-Tween. Membranes were reprobed with α-tubulin antibody (Sigma, final dilution 1/50,000) or ERK-2 (BD Transduction laboratories, final dilution 1/5,000) for normalization.

### Immunofluorescence staining and confocal microscopy

HepG2 and A549 cells were seeded at 50,000 cells/well while MCF-7 cells were seeded at 25,000 cells/well on glass coverslips in 24-well plates and 2 days later were incubated for different time with or without etoposide under normoxic or hypoxic conditions Immunofluorescence staining was performed as described in [20].

Primary antibodies were as follow : mouse anti-p53 (#05–224 Upstate) and rabbit anti-phospho-histone H2AX (#26079 Upstate). Alexa Fluor-568-conjugated anti-mouse or rabbit IgG antibody (Molecular Probes) were used at 1/1000 dilution.

### Nuclear protein extraction

Nuclear protein extractions in high salt buffer were prepared as previously described [37]. Briefly, cells seeded in 75 cm^2 ^flasks (Corning) were incubated with or without etoposide under normoxic or hypoxic conditions for 16 hours. At the end of the incubation, cells were rinsed with PBS containing 1 mM Na_2_MoO_4 _and 5 mM NaF and incubated on ice for 3 minutes with 10 ml cold Hypotonic Buffer (HB, 20 mM HEPES, 5 mM NaF, 1 mM Na_2_MoO_4_, 0.1 mM EDTA) and harvested in 500 μl HB containing 0.2% NP-40 (Sigma), a protease inhibitor cocktail (Roche) and phosphatase inhibitors (1 mM Na_3_VO_4_, 5 mM NaF, 10 mM p-nitrophenylphosphate, 10 mM β-glycerophosphate). Cell lysates were centrifuged 30 seconds at 13000 rpm and sedimented nuclei were resuspended in 50 μl HB containing 20% glycerol and protease/phosphatase inhibitors. Extraction was performed for 30 minutes at 4°C by the addition of 100 μl HB containing 20% glycerol, 0.8 M NaCl and protease/phosphatase inhibitors.

### DNA-binding assay

DNA-binding assays using TransAM ELISA kit (Active Motif) for detecting transcription factor DNA binding activity was performed according to the manufacturer's recommendations. Briefly, 10 μg of nuclear proteins were incubated for 2 hours in a 96-well plate coated with a double-stranded oligonucleotide containing the consensus sequence recognized by the transcription factor to be assayed (EPO enhancer sequence for HIF-1α). The transcription factor bound to DNA was detected using a specific primary antibody (rabbit anti-p53 (SC-6243 Santa Cruz) and mouse anti-HIF-1α (# 610958 Becton Dickinson)). Colorimetric reaction was then performed with a HRP-conjugated anti-rabbit IgG antibody and absorbance was measured at 450 nm in a spectrophotometer.

### Transient transfection and luciferase assay

Cell transfections were performed in 24-well plates (50,000 cells per well for HepG2 and A549, 40,000 cells per well for MCF-7) with SuperFect reagent (Qiagen). 1846 ng of the reporter plasmid pG13-Luc containing 13 copies of a p53-responsive promoter driving the expression of the luciferase gene [38] were co-transfected with 1154 ng of normalization vector (pCMVb vector coding for the β-galactosidase, Promega) in DMEM without serum for 8 hours. Cells were then directly incubated under hypoxia for 16 hours. After hypoxia incubation, β-galactosidase was assayed in parallel to the firefly luciferase activity, assayed using the Luciferase Reporter Assay System (Promega). Experiments were performed in triplicates. Results are expressed as means of the ratio between the firefly luciferase activity and the β-galactosidase activity.

### Gene expression analysis on DNA microarray

We used a low-density DNA array allowing the gene expression analysis for 123 genes related to apoptosis (DualChip^® ^human apoptosis, Eppendorf). Results using these reliable and validated arrays developed by Eppendorf were reported elsewhere [39-41]. The method is based on a system with two identical arrays on a glass slide and three identical sub-arrays (triplicate spots) per array. HepG2 cells cultured in 75 cm^2 ^flasks (Corning) were incubated for 16 hours with or without etoposide under normoxic and hypoxic conditions. At the end of the incubation, total RNA was extracted with the Total RNAgents extraction kit (Promega), quality was checked with a bioanalyzer (Agilent Technologies) and 20 μg were used for retrotranscription in the presence of biotin-11-dCTP (Perkin-Elmer) and Superscript II Reverse Transcriptase (InVitrogen), as described previously [39]. Hybridizations on the arrays were carried out as described by the manufacturer and reported previously [39]. Detection was performed with a cyanin 3-conjugated IgG anti-biotin (Jackson Immuno Research Laboratories). Fluorescence of hybridized arrays was scanned using a Packard ScanArray (Perkin-Elmer) at a resolution of 10 μm.

### Real time RT-PCR

After the incubation, total RNA was extracted using the Total RNAgent extraction kit (Promega). mRNA contained in 5 μg total RNA was reverse transcribed using SuperScript II Reverse Transcriptase (InVitrogen) according to the manufacturer's instructions. Forward and reverse primers for *MCL-1 *(*FP: *AAACGGGACTGGCTAGTTAAACAA, *RP: *TACTCCAGCAACACCTGCAAA), *BAK1 *(*FP: *CTTCGTGGTCGACTTCATGCT, *RP: *GGACCATTGCCCAAGTTCAG), *GPX1 *(*FP: *TTCCCGTGCAACCAGTTTG, *RP: *CCGGACGTACTTGAGGGAATT), *CDNK1A *(*FP: *CTGGAGACTCTCAGGGTCGAA, *RP: *CCAGGACTGCAGGCTTCCT), *aldolase *(*FP: *TGCGCAGGAGGAGTATGTCA, *RP: *AGGCGTGGTTAGAGACGAAGAG), *GADD45A *(*FP: *GGGCTGAGTGAGTTCAACTACATG, *RP: *GCTTCCTTCTTCATTTTCACCTCTT), *GAPDH *(*FP: *ACCCACTCCTCCACCTTTGAC, *RP: *GTCCACCACCCTGTTGCTGTA) and *RPL13A *(*FP: *CTCAAGGTCGTGCGTCTGAA, *RP: *TGGCTGTCACTGCCTGGTACT) were designed using the Primer Express 1.5 software (Applied Biosystem). Amplification reaction assays contained 1× SYBR Green PCR Mastermix (Applied Biosystem) and primers (Eurogentec) at the optimal concentrations. A hot start at 95°C for 5 minutes was followed by 40 cycles at 95°C for 15 seconds and 65°C for 1 minute using an ABI PRISM 7000 SDS thermal cycler (Applied Biosystem). *RPL13A *was used as the reference gene for normalization and mRNA expression level was quantified using the threshold cycle method.

## Competing interests

The author(s) declare that they have no competing interests.

## Authors' contributions

JPC, AS and KV carried out all the experiments, NN carried the immunofluorescence studies, MR participated in the design of the study, CM conceived of the study, participated in its design and coordination and helped to draft the manuscript. All authors read and approved the final manuscript.

## Supplementary Material

Additional file 1 Effect of hypoxia on the etoposide-induced DNA damage. The pictures show immunofluorescence stainings for DNA damage. A549, MCF-7 or HepG2 cells were incubated under normoxic or hypoxic conditions with or without etoposide (50 μM) for 1 (*A*) or 16 (*B*) hours. After the incubation, cells were fixed, permeabilized and stained for the phosphorylated form of the histone H2AX using a specific antibody (green). Nuclei were detected with To-Pro-3 (blue). Observation was performed using a confocal microscope with the photomultiplier constant. Click here for file

Additional file 2 Gene expression profiling in A549, MCF-7 and HepG2 cells incubated with or without etoposide under normoxic or hypoxic conditions. Data are given for the 62 genes for which there was a significant variation in expression for at least one of the conditions. Cells were incubated under normoxic (N) or hypoxic (H) conditions with or without etoposide (e, 50 μM) for 16 hours before RNA extraction, reverse-transcription and cDNA hybridization, as described in Materials and Methods. Each value is the average of three ratio values calculated from three independent experiments ± 1 S.D. Mean ratios indicate a fold-increase or decrease in gene expression. They are highlighted in blue if statistically non significant, in yellow for quantitative data and in green for qualitative data, given with + or - signs (according to the inserted table). Click here for file
