# Supplementary figures and images for: Differential effects of hypoxia on etoposide-induced apoptosis according to the cancer cell lines
Source: Mol Cancer. 2007 Sep 26;6:61. doi: 10.1186/1476-4598-6-61 (PMC2099441; doi:10.1186/1476-4598-6-61)

## Slide 1
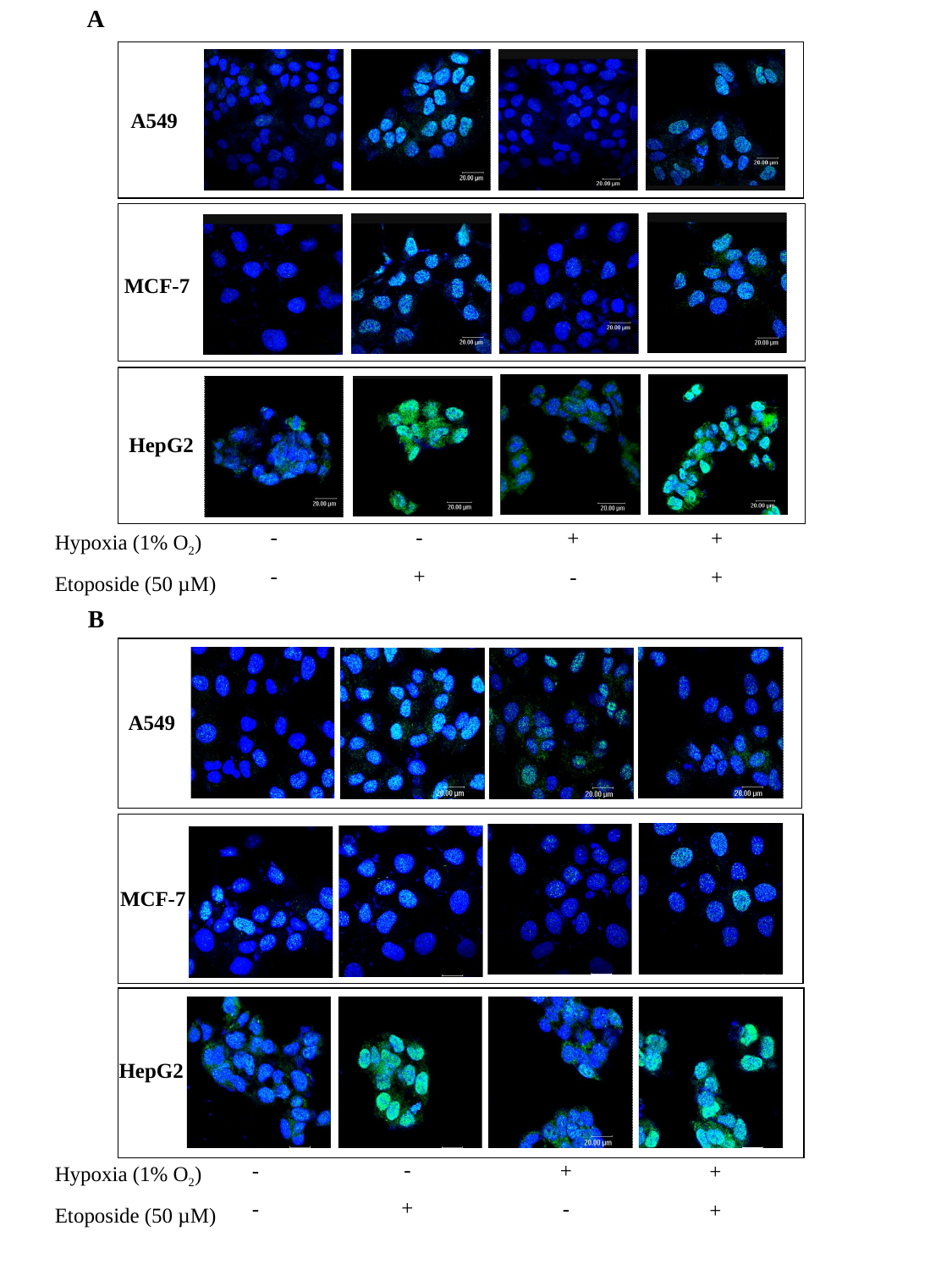

A
A549
MCF-7
HepG2
-
-
-
+
+
-
+
+
Hypoxia (1% O2)
Etoposide (50 µM)
B
A549
MCF-7
HepG2
-
+
-
-
+
-
+
+
Hypoxia (1% O2)
Etoposide (50 µM)

Supplement: Additional file 1 — Effect of hypoxia on the etoposide-induced DNA damage. The pictures show immunofluorescence stainings for DNA damage. A549, MCF-7 or HepG2 cells were incubated under normoxic or hypoxic conditions with or without etoposide (50 μM) for 1 (A) or 16 (B) hours. After the incubation, cells were fixed, permeabilized and stained for the phosphorylated form of the histone H2AX using a specific antibody (green). Nuclei were detected with To-Pro-3 (blue). Observation was performed using a confocal microscope with the photomultiplier constant. [file 1476-4598-6-61-S1.ppt]
